# Supplementary material for: Clonal expansion of mitochondrial DNA deletions is a private mechanism of aging in long‐lived animals
Source: Aging Cell. 2018 Jul 24;17(5):e12814. doi: 10.1111/acel.12814 (PMC6156498; doi:10.1111/acel.12814)
Supplement: Supplementary file 1 [file ACEL-17-e12814-s001.docx]

**Title: Clonal expansion of mitochondrial DNA deletions is a private mechanism of ageing in long-lived animals**

Lakshmi Narayanan Lakshmanan^3,4,*^, Zhuangli Yee^2,*^, Li Fang Ng^1^, Rudiyanto Gunawan^3,4^ , Barry Halliwell^2^ and Jan Gruber^1,2^

**Supporting Information**

Table of Contents

[Coverage by 236 primer pairs 2](#_Toc514999135)

[Figure S1 3](#_Toc514999136)

[Figure S2. 4](#_Toc514999137)

[Figure S3. 5](#_Toc514999138)

[Computational procedure for mutant fraction estimation 5](#_Toc514999139)

[Figure S4. 7](#_Toc514999140)

[Figure S5 8](#_Toc514999141)

[Random mutation capture assay: plate design and controls 9](#_Toc514999142)

[Figure S6. 9](#_Toc514999143)

[Table S1 11](#_Toc514999144)

[Table S2. 12](#_Toc514999145)

[Table S3. 14](#_Toc514999146)

[Table S4. 15](#_Toc514999147)

[Table S5. 17](#_Toc514999148)

# Coverage by 236 primer pairs

PCR based methods generally suffer from PCR amplification bias, where lengthy deletions with breakpoints close to the primers are preferentially amplified more than other short length deletions. Net result of such a bias is that only a subset (within *in vivo* deletion spectra) of deletions close to primer positions get detected. To overcome this bias and obtain a comprehensive sample from *in vivo* spectrum of mtDNA deletions, we designed 236 distinct primer pairs, spanning a 9000 bp region between 1800 and 10800 positions in worm mtDNA (Fig. 2 Main text). This set of 236 distinct primer pairs were obtained by the combination of 12 forward and 36 reverse primers (after excluding the unfeasible combinations). Positions, length and sequences of the primers and probes are provided in the Table S3 (Supporting information). We designed the locations of the primers to achieve two objectives, (1) any possible deletion breakpoint pair between 1800-10800 bp worm mtDNA positions should get amplified by at least 1 primer pair. (2) Deletion breakpoints within a short stretch of mtDNA regions should get amplified primarily by only one primer pair. This objective was made possible by setting a short duration for PCR extension step. The PCR protocol used for the capture experiments in our mutation capture assay was designed so that it can only amplify fragments up to a length of 1500 bp (see Fig. S3 below). To determine the ability of the 236 primer pairs to detect any possible deletion mutation that can happen between positions 1800-10800 in *C. elegans* mtDNA, we used a uniform random number-based sampling method to generate 100,000 random pairs of breakpoints with deletion lengths uniformly distributed between a specified minimum and maximum deletion length. Primer pairs that are positioned within 1000 bp will always result in the wildtype mtDNA amplification and hence are not included for the analysis. For each randomly generated deletion, we then determined if any set of PCR primers could in principle amplify the deletion based on the calculated amplicon length for each of the primer pairs. If a deletion could be amplified by at least 1 primer pair with an amplicon length < 1000 bp, we consider this deletion as ‘detectable’. Using this procedure, we calculated the percentage of all deletions that could be successfully detected by at least one of the primer pairs. Using this analysis, we found that 95% of possible deletions (deletion length ≥ 500 bp) were detectable using our capture protocol (Fig. S1). Typically, short deletions (deletion length < 250 bp) escape detection during the mutation capture assay.

Figure S1. **Coverage by primer pairs**


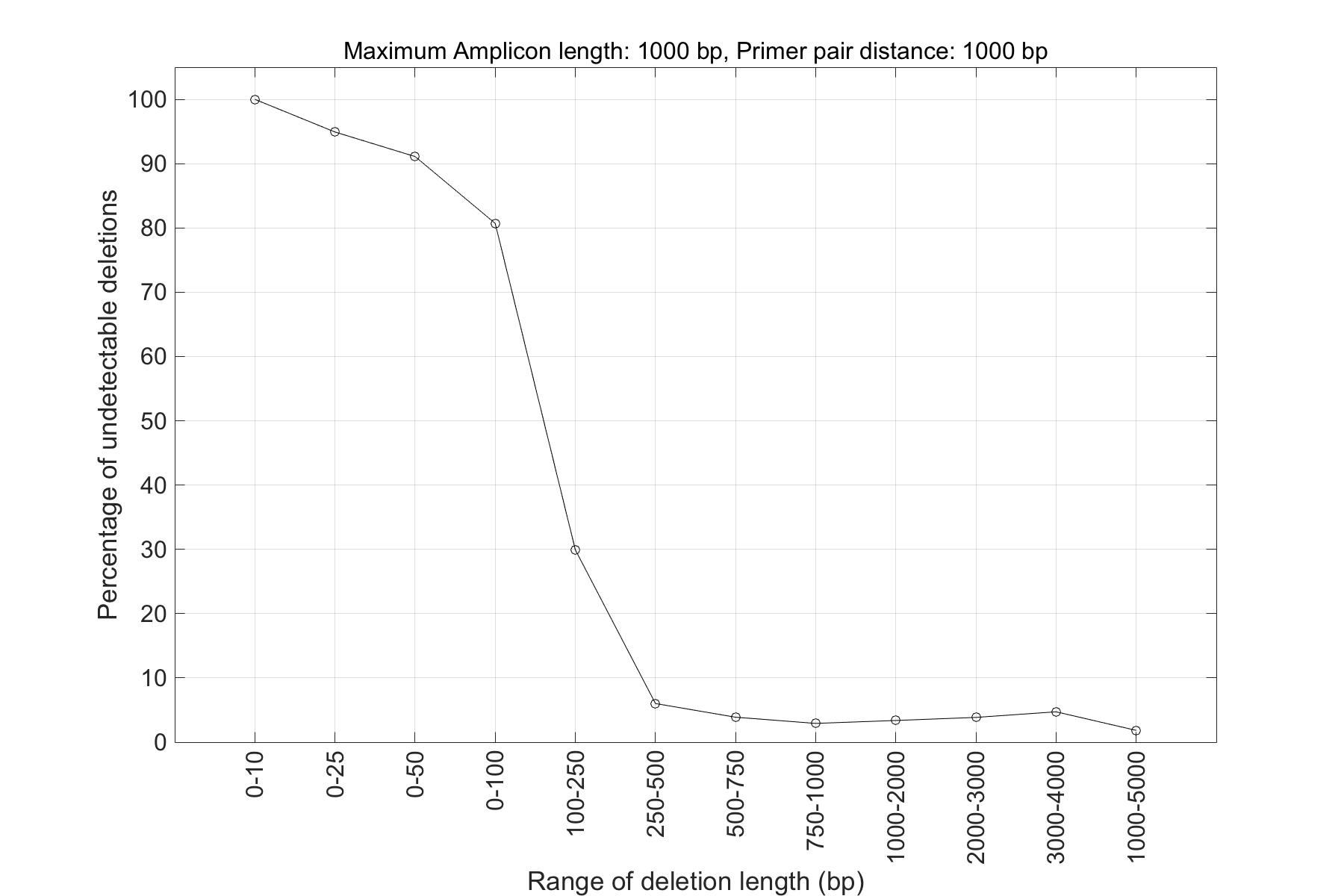


**Figure S1.** Percentage of randomly generated deletion breakpoints that escaped the detection by all of the primer pairs under the PCR conditions used in our study. Our primer design successfully detected 95% of possible deletions with a deletion length ≥ 500 bp between positions 1800 and 10800 bp in *C. elegans* mtDNA. Primer pairs (among the 236 pairs) which are positioned < 1000 bp apart in the wild type mtDNA sequence are able to amplify the WT sequence. These primers were used as positive control but not included in the analysis.

Figure S2. Artificially synthesized mutant sequence

GAGCGTCATTTATTGGGAAGAAGACAAAATCGTCTAGGGCCCACCAAGGTTACATTCCCGGGATTAGCACAAGCTAACTTAATTTAGAATGTATCACTTACAATGATGGGGTCGCGCGCCGGTCTATAGTAAAAGTGTTCGGCCTTAAGTAGGACCGCCAGATGCGCGAATATTGACCCTATCGCGAGAAGCCCGTTTCTTATCGGGCCACTACGGGGCGTATACCAGGGCGGaCAATGAGAATACATAGGaGCGCTTACTTTATTTGGTTTTGCAGCTGTAATTAGCGGTATCGTAAGAAAATCAAAATATGGTATAAcTccGGggATCCGTGCTAGAAGACAgAGAATcTCTTAcGAAATcGCTTTcTCTTTATAcGTTTTGTGTATcATcATcCATAATAATGTTTTcAATTTcGTTTCAATcTAACCAGGCCGGCGCGCCGGTAGTATTTATATTTATTATCTCATCTACTGCCCGAGATGACTAGGTCAATGCACTGTAGAAGACCCGTTTTAAGGTCCGGGAGGTTTTTATTATAGTATTTACTGGGCaCCCGCCTGTTTCGCGCCGAGTATGTTTAGCGCGGCCAGTAGTTATTCTCGCTCGGGCGCCAGGTGCTTTACGTAAAGTTTAAAATTATGTTTTTATTTGTTAGATTATTTATATTTATTTTTAAATGACAACGTgTAgTTcTTgTTCTAgTTcCTTTTgTTgTTcCAATTcTAgGACCTTTATTTcCTTTTgTTcATTTTGGTgTAGCTTATTTAgTAcTGTTTgTTcTTgTAAGAAGTAAATTGTTATTTgAATAATATTATTAcTCTTTTGCTTGAGGTAAGGATATTGCT

**Figure S2.** The artificially synthesized mutant has the sequences for primers F1, P1 and R16, and parts of the mtDNA: bp position 1838 to 1912, 69 to 102, 2105 to 2261, and 5453 to 5524. This artificial mutant is used for positive control and for serial dilution experiments using the primer pair (F1, R16).

Figure S3. Serial dilution experiments


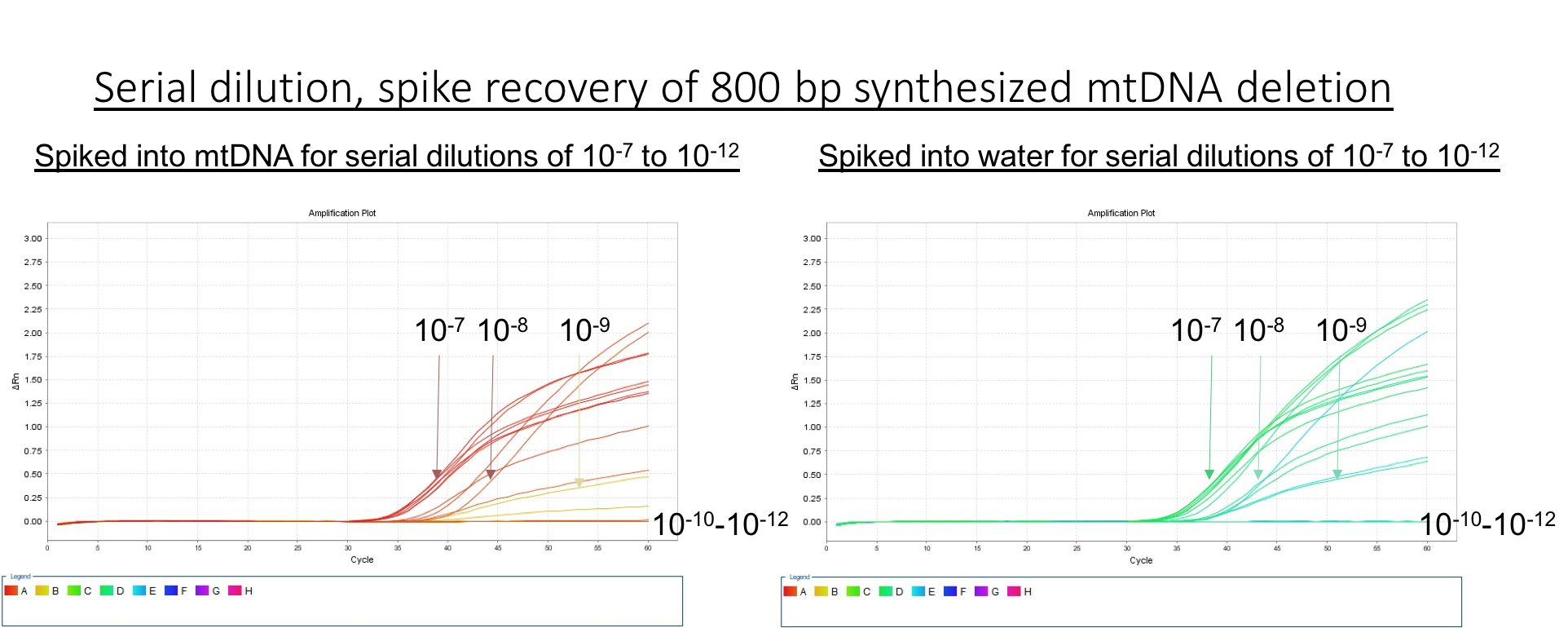


**Figure S3.** Random mutation capture assay with serially diluted samples of artificially synthesized mutant sequence. This synthetic mutant results in 800 bp long amplicon using the primers F1 and R16. For each dilution, spike sample was added to either water (6 wells) or wild type mtDNA (6 wells). At a dilution of 10^-9^ amplification becomes stochastic with only 2 out of the 6 wells amplifying for both water and mtDNA samples.

# Computational procedure for mutant fraction estimation

Experimentally, mtDNA is extracted from worm homogenate, diluted and the aliquoted to PCR wells. These 3 steps, extraction, dilution and aliquoting can be mathematically described as random sampling of mtDNA molecules from a population without replacement (e.g. extraction: N_mtDNA, extract_ mtDNA molecules were randomly sampled without replacement from N_mtDNA, homogenate._ Homogenate contains N_W, homogenate_ wildtype and N_M, homogenate_ mutant mtDNA molecules). For each of these three sampling steps, the number of mutant mtDNA molecules in the random sample was calculated using hypergeometric sampling.

For each experiment, using the experimentally used values for worm count in homogenate and extract volume, we generated *in silico* worm homogenate samples with a broad range of mutant fractions (10^−10^ to 10^−1^ (mutant mtDNA count / total mtDNA count)). *In silico* homogenate mtDNA samples are generated for a simple scenario with a single type of mutant. Distinct deletion mutations differ from each other in their breakpoint positions. However, for mutant fraction estimation, the sampling statistics and primer pair – breakpoint matching probability values are the same for each distinct deletion. For each of these homogenate samples, we simulated replicate mutation capture assays identical to the experiments. For each mutation capture experiment, we obtained the number of mutant mtDNA molecules in the 236 PCR wells using sequence of hypergeometric sampling steps. A deletion mutation with a specific breakpoint pair is expected to be amplified by 1/236 primer pairs. Hence the probability of a single mutant molecule to meet its matching primer pair is 1/236. If there are n_mutant_ of such mutant mtDNA molecules among the 236 well samples, then the probability that the mutant would get amplified (p_amplification_) is n_mutant_ * (1/236). For each set of 236 well samples, we draw a uniform random number between 0 and 1. If p_amplification_ > uniform random number, we assign a positive amplification for the mutant. In this manner, we obtained an *in silico* calibration curve relating the observed primer pair amplification fraction among replicates and the mutant fraction (burden) in the homogenate (Supporting Figure S4). Mutant fractions (mutant mtDNA count / total mtDNA count) lower than 10^−7^ rarely get detected and mutant fractions above 10^−4^ gets detected 100%. The dynamic range for primer pair amplification count exists between 10^−7^ and 10^−4^ (Supporting Figure S4).

For a cohort with n_replicate_ mutation capture experiments, there are (n_replicate_ + 1) different outcomes are feasible. For a cohort with 5 replicates, the potential primer pair amplification count values include {0,1,2,3,4,5} (Supporting Figure S4). For each possible outcome, we extracted the distribution of mutant fraction values from the calibration curve. For each deletion mutation, we used its primer pair amplification count to assign a mutant fraction value. This mutant fraction value is obtained by inverse sampling procedure using the cumulative density function of the primer pair amplification count. Finally, we summed up the estimated mutant fraction values of all the deletions in the cohort to obtain the total mutant fraction value. As hypergeometric sampling is a probabilistic method, we repeated this analysis 1000 times to obtain the median and 95% confidence intervals of total mutant fraction for each age group. Original values obtained were applicable only to the 9000 bp region covered by the primer pairs. Hence, we adjusted to mutant fraction value to the whole mtDNA length.


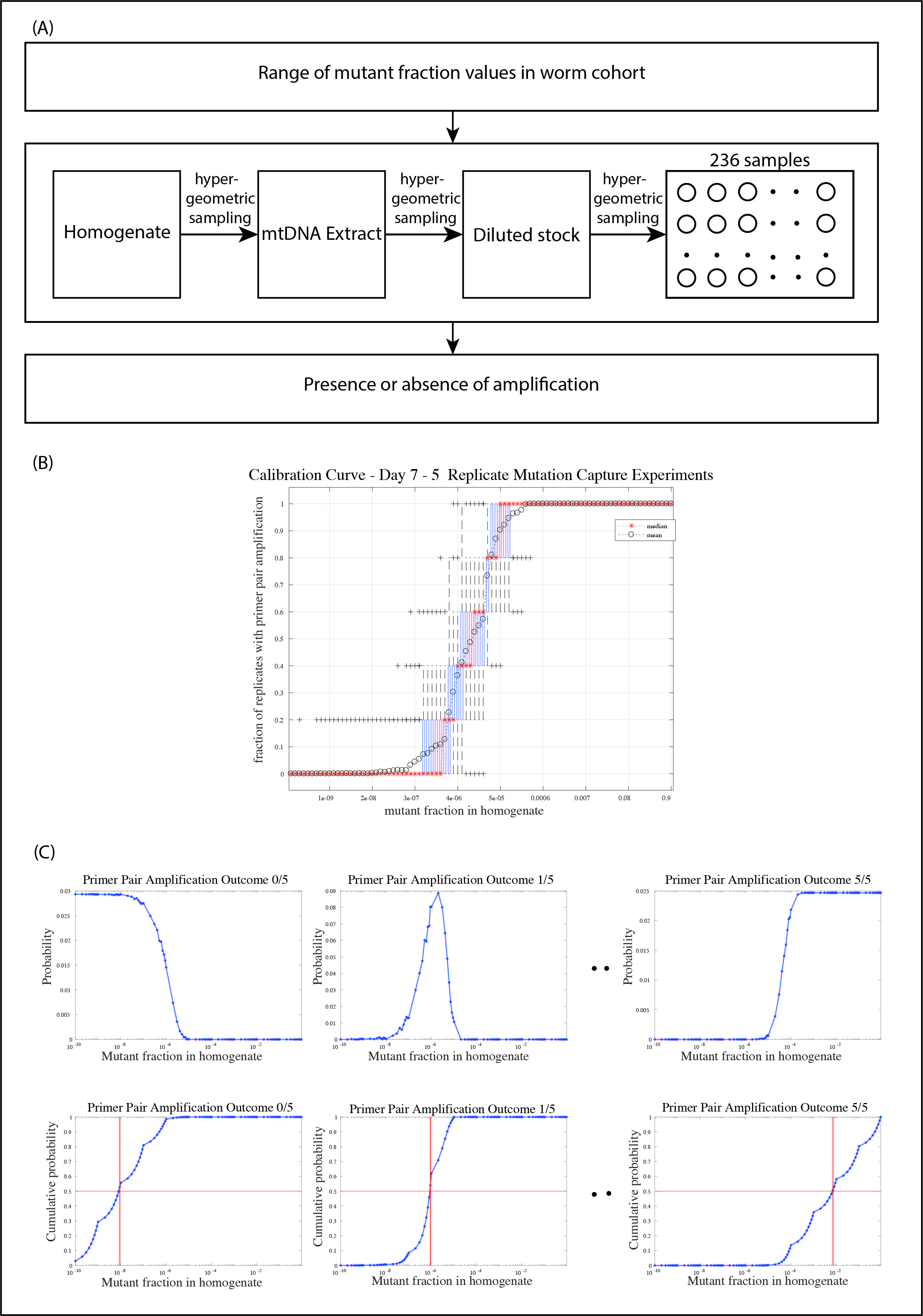


Figure S4. Estimation of mtDNA deletion mutant fraction. (A) We simulated the mtDNA extraction and mutation capture procedures as a series of hypergeometric sampling events. For each hypothetical mutant fraction (virtual deletion burden), we repeated the entire *in silico* sampling experiment (exactly mirroring the actual steps carried out in the experiment) for 1000 times. Using this procedure, for each hypothetical mutant fraction we obtained an estimate of the distribution of amplification counts (i.e. the number of PCR wells amplified per experiment) that would be expected to be observed for that mutant fraction. (B) By repeating this procedure for a wide range of plausible virtual mutant fractions, we determined a calibration curve relating the number of expected amplification events (wells containing at least one mtDNA deletion) during the mutation capture experiments with the homogenate mutant fraction. (C) The calibration curve provides the mutant fraction distribution associated with a specific outcome in the mutation capture assay. For example, if a primer pair amplified 1 out of 5 replicates, the mutant fraction of that specific deletion is expected to be between 1e-8 and 1e-4. During quantification, for each primer pair with 1/5 amplification, we use inverse sampling procedure to randomly sample a mutant fraction value from the probability distribution function obtained from the calibration curve.


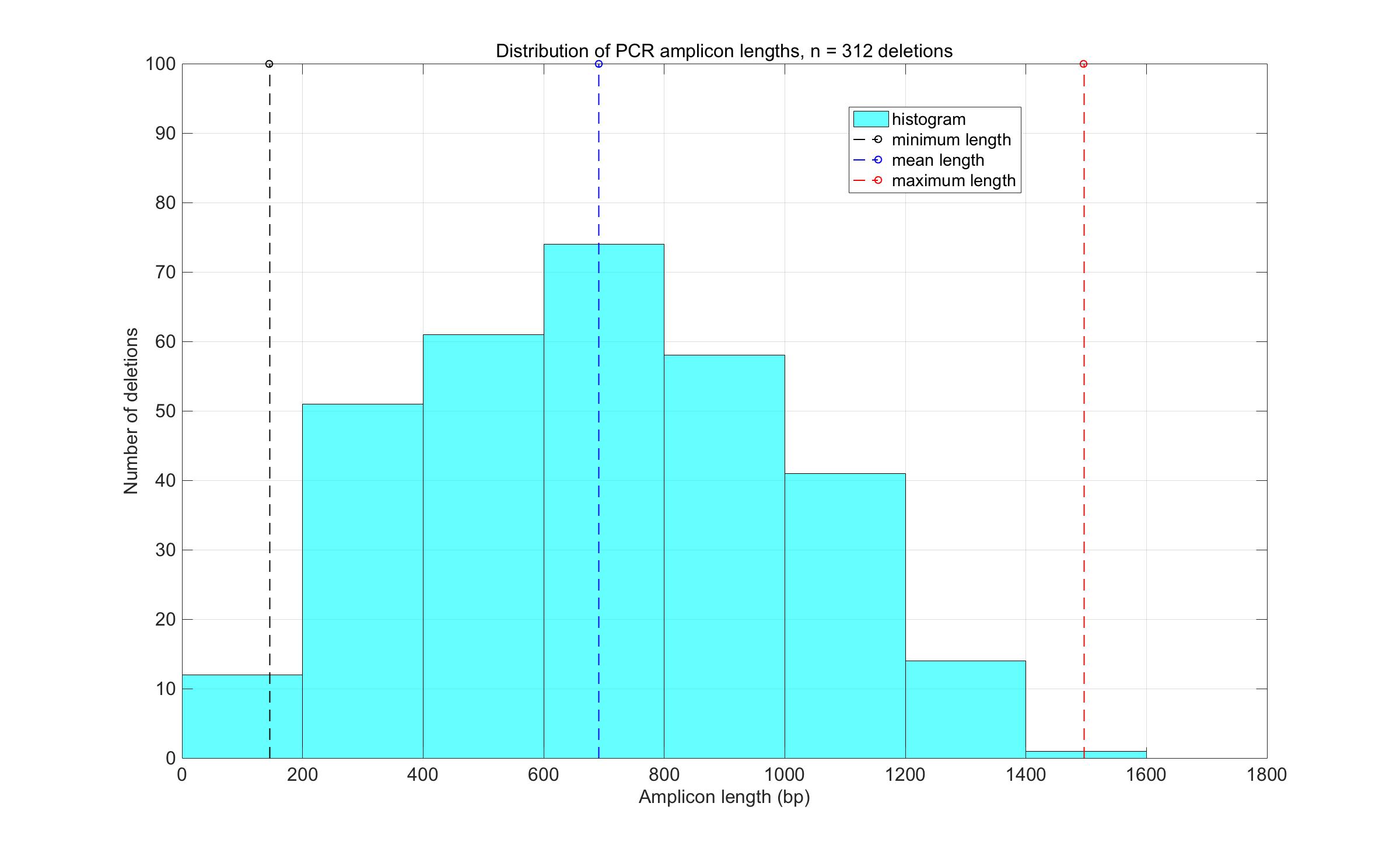


Figure S5. Distribution of the amplicon lengths of deletions detected by mutation capture assay. Consistent with the design of the mutation capture assay (primer location), the amplicon lengths are distributed below 1500 bp length.

# Random mutation capture assay: plate design and controls

In each repeat of the random mutation capture assay, we used 236 distinct primer pairs to capture mtDNA deletions present in mtDNA samples extracted from worm cohorts. Hence, each single run of the assay involved three ’96-well’ plates. Figure S6 shows the organization of wells with distinct primer pairs and the location of positive and negative control wells on these three 96-well plates.

*
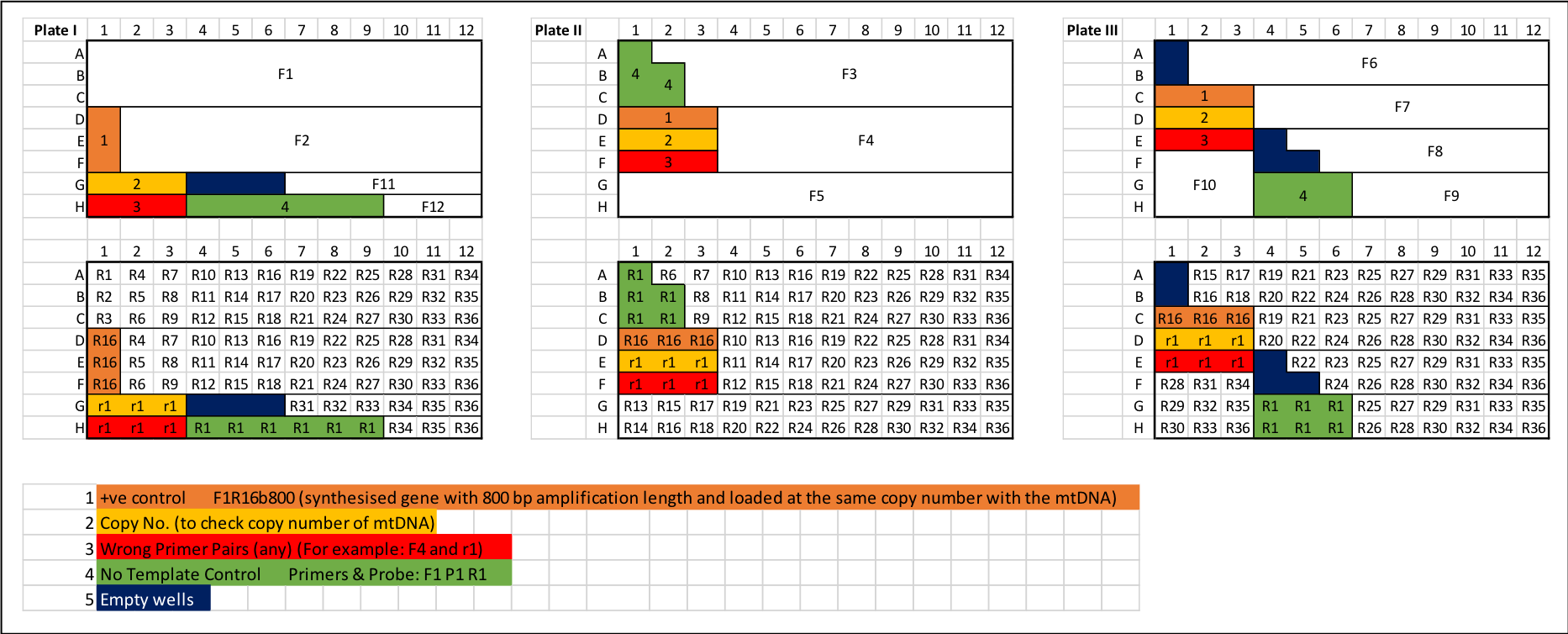
*

Figure S6. Plate design for wells with 236 distinct primer pairs and control samples in the three 96-well plates used for each random mutation capture assay.

On each of these 3 plates, we had four types of controls. They were:

1. Positive mtDNA deletion control: For positive control wells, we used an artificially synthesized DNA deletion construct resulting in a 800 bp long amplicon using the F1-R16 primer pair (see Fig.S2). Three wells were used on each plate for this positive control. In all cases (all plates of all repeats of the assay), this positive control amplified, and amplification resulted in the expected amplicon.
2. Copy number WT mtDNA positive control: A short fragment of wild type mtDNA was used as a copy number control. Primer pair F1-r1 was used, resulting in a 71 bp amplicon. Three wells of each plate were used for this copy number control in each experiment. The copy number control worked successful in each case and Cts were consistent between experiments.
3. Incompatible primer pair negative control: Incompatible primer pairs refer to the pairs of forward and reverse primers which, due to their reversed position in the mtDNA template, cannot generate any amplicon, irrespective of the presence of any mtDNA deletion. Three wells were used on each plate for incompatible primer pairs for each experiment. As expected, these wells never resulted in amplification during any of our experiments.
4. No template negative control (NTC): In these control wells, water was added instead of mtDNA. Hence, these wells are not expected to give rise to any amplification, unless due to contamination or carry over. 5-6 wells per plate were allocated for NTC controls and the F1-R1 primer pair was used for these wells. In rare instances, we did detect an amplification after 35 cycles. In each case, samples from these wells were run on agarose gel and typically resulting in only a faint band with low DNA concentration, possibly due to DNA contamination. Sequencing attempts of these rare faint bands failed to reveal the presence of mtDNA sequences.

Table S1. **Experimental Design**

| Age group | Cohort number | Number of replicates  (mutation capture assays) | Number of deletions detected | Average deletions per replicate |
| --- | --- | --- | --- | --- |
|  |  |  |  |  |
| Day-4 | 1 | 4 | 43 | 10.75 |
|  | 2 | 3 | 21 | 7.00 |
|  | 3 | 4 | 37 | 9.25 |
| Day-7 | 1 | 1 | 14 | 14.00 |
|  | 2 | 1 | 7 | 7.00 |
|  | 3 | 1 | 9 | 9.00 |
|  | 4 | 1 | 11 | 11.00 |
|  | 5 | 1 | 14 | 14.00 |
| Day-10 | 1 | 1 | 4 | 4.00 |
|  | 2 | 10 | 148 | 14.80 |
|  | 3 | 1 | 4 | 4.00 |

**Table S1.** Detailed information about the experimental design for mutation capture assay provided in Table 1 in main text. We used at least three independent cohorts for each age group. Dependent on DNA yield, we then carried out between 1 and 4 repeat mutation capture assays per cohort. For one of the day-10 cohorts we instead carried out a total of ten repeats of the mutation capture assay using a single tube of mtDNA extract from this single day-10 cohort. This was done to permit comparison of in-between-cohort with within-cohort repeat detection rate of deletions.

Table S2. **Results from replicate mutation capture experiments**

| Age Group | Total replicate experiments | Number of amplifications within replicates | Number of primer pairs |
| --- | --- | --- | --- |
| Day-4 | 11 | 0 | 173 |
|  |  | 1 | 37 |
|  |  | 2 | 19 |
|  |  | 3 | 4 |
|  |  | 4 | 3 |
| Day-7 | 5 | 0 | 192 |
|  |  | 1 | 36 |
|  |  | 2 | 5 |
|  |  | 3 | 3 |
| Day-10 | 12 | 0 | 152 |
|  |  | 1 | 40 |
|  |  | 2 | 22 |
|  |  | 3 | 10 |
|  |  | 4 | 6 |
|  |  | 6 | 2 |

**Table S2.** Results from mutation capture experiments. We collected at least three biologically independent cohorts for each age group. Dependent on DNA yield, we then carried out between 1 and 4 repeat mutation capture assays per cohort. For one of the day-10 cohorts we instead carried out a total of ten repeats of the mutation capture assay. Taken together, we did 11, 5 and 12 replicate mutation capture assays for days 4,7 and 10, respectively. Each replicate experiment involved 236 primer pairs. For each age group, we calculated the number of times each primer pair detected an amplification (e.g. number of times each primer pair detected an amplification during the 11 replicate experiments done for day-4). We binned the primer pairs based on the number of times they detected amplification (the number of times a given well showed amplification with sequencing confirming an actual mtDNA deletion). For example, the first row indicates 173 primer pairs (out of 236 pairs) detected 0 mutants, 37 detected exactly 1 mutation, 19 detected 2, 4 detected 3 and 3 detected 4 during the 11 replicate mutation capture assay experiments performed for all day-4 cohorts. We subsequently used this binned data from mutation capture experiments to estimate the mutant fraction in worm homogenates using sampling statistics and Monte Carlo sampling.

For most primer pairs of the day-10 cohort we ran 10 repeats of the mutation capture experiment (10 wells for each primer pair, see Table S1). However, for a small number of primer pairs in the day-10 cohort 2, fewer than10 assays were performed due to sample loss or problems during the PCR run of some repeats. These differences in the replicate counts, for specific primer pairs, has been correctly accounted for during mutant fraction calculation.

Table S3. Repeatedly detected mtDNA deletions


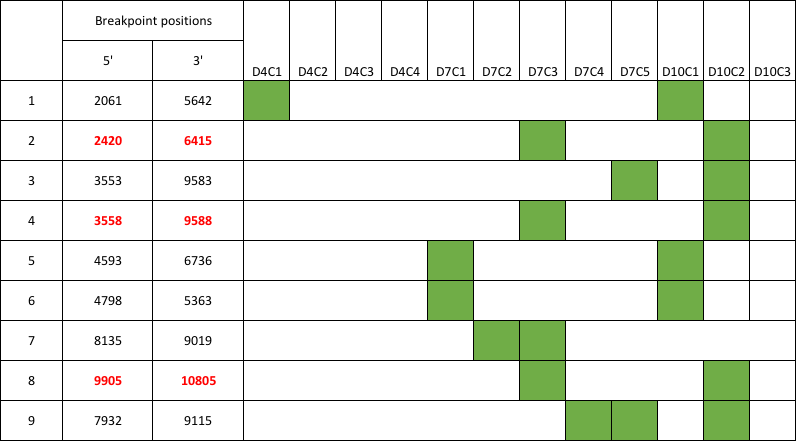


**Table S3.** mtDNA deletions repeatedly detected in multiple cohorts. Nine deletions were detected in more than 1 cohort. For each deletion, the cohorts that contained the deletion is highlighted in green.

Table S4. **Primer positions and sequence information**

| Forward Primers |  | Sequence | Start base | End base | Length |
| --- | --- | --- | --- | --- | --- |
| **F1** | **F1** | GAGCGTCATTTATTGGGAAGAAGA | 1838 | 1861 | 24 |
| **F750** | **F2** | TCGTAGTTCATACCCTCGTTATCG | 2524 | 2547 | 24 |
| **F1500** | **F3** | GTGCATTTGTTTTAAGCGCAA | 3325 | 3345 | 21 |
| **F2250** | **F4** | TTCTCATTGAGGGAAATTTTTAAAT | 4075 | 4099 | 25 |
| **F3000** | **F5** | CAAATAAGATTTTGAGCAGCAGTAGTT | 4903 | 4929 | 27 |
| **F3750** | **F6** | CAATTTTGCGTGCTATTCCAA | 5327 | 5347 | 21 |
| **F4500** | **F7** | GTCTTGTGTGGTGGTTTGTTCTTAG | 6281 | 6305 | 25 |
| **F5250** | **F8** | TTAGGCACAGCGGGATTTTT | 7109 | 7128 | 20 |
| **F6000** | **F9** | AAAATATCAAGGAGGATTGGCAGT | 7859 | 7882 | 24 |
| **F6750** | **F10** | CCAAGAACTGGAGGTAATCCTCTT | 8529 | 8552 | 24 |
| **F7500** | **F11** | TTACCCTGATGTTTATTCGGTATGA | 9191 | 9215 | 25 |
| **F8250** | **F12** | AGGTGAACCACGTTTATTAGAAGTTG | 10047 | 10072 | 26 |
| Probes |  |  |  |  |  |
| **P1** |  | AAAATCGTCTAGGGCCCAC | 1863 | 1881 | 19 |
| **P2** |  | TTGTGTTTTTACGCAGTTAT | 2606 | 2625 | 20 |
| **P3** |  | ACTAACGAGTTCATAAAGCAA | 3359 | 3379 | 21 |
| **P4** |  | ATCTGTATTGGCATTTAGAT | 4143 | 4162 | 20 |
| **P5** |  | TCCAATTTGAGGGCCAACT | 4953 | 4971 | 19 |
| **P6** |  | TCTTAGGGGTAATTGCTTTAT | 5354 | 5374 | 21 |
| **P7** |  | TTGTTGATGTAGTGTGATTAT | 6390 | 6410 | 21 |
| **P8** |  | TAGGTAGTTTAAGATTTGTACACAAC | 7137 | 7162 | 26 |
| **P9** |  | ATCATAAAGATATCGGAACTCT | 7900 | 7921 | 22 |
| **P10** |  | TTTTGGTCATCCTGAAGT | 8576 | 8593 | 18 |
| **P11** |  | TTGCCTCTTATGGTTCTATT | 9223 | 9242 | 20 |
| **P12** |  | TAATCGTTGTGTTATTCCTT | 10074 | 10093 | 20 |
| Reverse Primers |  |  |  |  |  |
| **R1** | **r1** | TGTGCTAATCCCATAAATGTAACCTT | 11887 | 11912 | 26 |
| **R0250** | **R1** | GGATGGCCCCAATTATACCA | 11627 | 11646 | 20 |
| **R0500** | **R2** | GCCCGGTTAAGTTCAGCAAT | 11483 | 11464 | 20 |
| **R0750** | **R3** | CGATAACGAGGGTATGAACTACGA | 11248 | 11271 | 24 |
| **R1000** | **R4** | AAGTAAATTCAACCATTCCACAAGG | 10909 | 10933 | 25 |
| **R1250** | **R5** | AACGCACTGTTAAAGCAAGTGG | 10741 | 10762 | 22 |
| **R1500** | **R6** | AAACGAGCAGGATTTAACCTAGAAC | 10378 | 10402 | 25 |
| **R1750** | **R7** | AAATTCTGGTATATCTCTTGCTGCTT | 10230 | 10255 | 26 |
| **R2000** | **R8** | AACAAAATATACACGGATCTTAACCAA | 9992 | 10018 | 27 |
| **R2250** | **R9** | ATTTAAAAATTTCCCTCAATGAGAA | 9696 | 9720 | 25 |
| **R2500** | **R10** | TTCTCCCAACTGGAACTTTACCTT | 9469 | 9492 | 24 |
| **R2750** | **R11** | TCGGGCGTATAATAAAATGCTAA | 9128 | 9150 | 23 |
| **R3000** | **R12** | AACTACTGCTGCTCAAAATCTTATTTG | 8866 | 8892 | 27 |
| **R3250** | **R13** | TCACCGTGGCAATATAACCTAGA | 8660 | 8682 | 23 |
| **R3500** | **R14** | ATCACTCTGGAACAATATGAACTGG | 8487 | 8511 | 25 |
| **R3750** | **R15** | AAAGGTCTTAAAATTGTAAAAGGGTCT | 8255 | 8281 | 27 |
| **R4000** | **R16** | AGCAATATCCTTACCTCAAGCAAAA | 7941 | 7965 | 25 |
| **R4250** | **R17** | AACTAAGTGCATTCCAAAAGGTGA | 7758 | 7781 | 24 |
| **R4500** | **R18** | CTAAGAACAAACCACCACACAAGAC | 7490 | 7514 | 25 |
| **R4750** | **R19** | AAAAATAGTCCGCCTCATGAAAA | 7171 | 7193 | 23 |
| **R5000** | **R20** | TTTCAATTTGAGAACCATAACCTAGAA | 6941 | 6967 | 27 |
| **R5250** | **R21** | AAAAATCCCGCTGTGCCTAA | 6667 | 6686 | 20 |
| **R5500** | **R22** | TATACGCCTCCCAGATGTATGATAA | 6384 | 6408 | 25 |
| **R5750** | **R23** | GAAGGTGGTACACCCCTATTTGA | 6292 | 6314 | 23 |
| **R6000** | **R24** | ACTGCCAATCCTCCTTGATATTTT | 5913 | 5936 | 24 |
| **R6250** | **R25** | ATCAGTTACCAAAACCACCGATTA | 5683 | 5706 | 24 |
| **R6500** | **R26** | TTAACCCTGCTGCATGTAAACTAAA | 5464 | 5488 | 25 |
| **R6750** | **R27** | AAGAGGATTACCTCCAGTTCTTGG | 5243 | 5266 | 24 |
| **R7000** | **R28** | ATAGTAGCAGCCGAAAAATAAGCAC | 5001 | 5025 | 25 |
| **R7250** | **R29** | GAAAATCCCAAAAACAGCTCCT | 4760 | 4781 | 22 |
| **R7500** | **R30** | TCATACCGAATAAACATCAGGGTAA | 4580 | 4604 | 25 |
| **R7750** | **R31** | AAAAACCAATACCATTTAATTTGCAA | 4318 | 4343 | 26 |
| **R8000** | **R32** | TCCCAACAATAAACTACAATTAAACCT | 4045 | 4071 | 27 |
| **R8250** | **R33** | CAACTTCTAATAAACGTGGTTCACCT | 3723 | 3748 | 26 |
| **R8500** | **R34** | GGTATAAAACTATGATTTGCTCCACAA | 3515 | 3541 | 27 |
| **R8750** | **R35** | CAGTTATCACAAAGGTCGACATATCA | 3235 | 3260 | 26 |
| **R9000** | **R36** | CCTCACGCTAAGACTGCCATT | 2936 | 2956 | 21 |

Table S5. Parameter values used for stochastic model simulations

Detailed description of model equations and parameters (e.g. V-max value calculations) could be found in our original model publication (Poovathingal *et al.* 2009).

**References:**

Poovathingal SK, Gruber J, Halliwell B, Gunawan R (2009). Stochastic Drift in Mitochondrial DNA Point Mutations: A Novel Perspective Ex Silico. *PLOS Computational Biology*. **5**, e1000572.
