# Supplementary material for: Clonal expansion of mitochondrial DNA deletions is a private mechanism of aging in long‐lived animals
Source: Aging Cell. 2018 Jul 24;17(5):e12814. doi: 10.1111/acel.12814 (PMC6156498; doi:10.1111/acel.12814)
Supplement: Supplementary file 2 [file ACEL-17-e12814-s002.docx]

**Title: Clonal expansion of mitochondrial DNA deletions is a private mechanism of ageing in long-lived animals**

Lakshmi Narayanan Lakshmanan^3,4,*^, Zhuangli Yee^2,*^, Li Fang Ng^1^, Rudiyanto Gunawan^3,4^ , Barry Halliwell^2^ and Jan Gruber^1,2^

**Supporting Document 2 – Breakpoints Data**

**Day 4 Cohort 1**

**Day 4 Cohort 2**

**Day 4 Cohort 3**

**Day 7 Cohort 1**

**Day 7 Cohort 2**

**Day 7 Cohort 3**

**Day 7 Cohort 4**

**Day 7 Cohort 5**

**Day 10 Cohort 1**

**Day 10 Cohort 2**

**Day 10 Cohort 3**
